# Effects of Ginger (*Zingiber officinale*) and Locust Bean (*Parkia biglobosa*) Supplementation on Hepatic Caspase‐3 and Bcl‐2 Expression in Wistar Rats

**DOI:** 10.1002/fsn3.71564

**Published:** 2026-03-26

**Authors:** Basiru Olaitan Ajiboye, Olawale Razaq Ajuwon, Aminu Momoh Jimoh, Mustapha Taiwo Olawale, Iyanuoluwa Peter Adesuyi, Iyanuoluwa Temilade Fasugba, Precious Eseose Agboinghale, Mary Abiola Okesola, Adetutu Omolola Ojelabi, Ahmed Olatunde, Temitope Olawale Jeje, Taoheed Olawale Bello, Babatunji Emmanuel Oyinloye, Kasim Sakran Abass

**Affiliations:** ^1^ Phytomedicine and Molecular Toxicology Research Laboratory, Department of Biochemistry Federal University Oye‐Ekiti Oye Ekiti State Nigeria; ^2^ Redox Biology and Phytomedicine Research Laboratory, Department of Biochemistry Federal University Oye‐Ekiti Oye Ekiti State Nigeria; ^3^ Department of Chemistry and Biomolecular Sciences, Faculty of Science University of Ottawa Ottawa Ontario Canada; ^4^ Department of Chemistry and Biochemistry Caleb University Lagos Nigeria; ^5^ Department of Medical Biochemistry Abubakar Tafawa Balewa University Bauchi Nigeria; ^6^ Biochemical Immunology and Phytomedicine Laboratory, Department of Biochemistry Federal University Oye‐Ekiti Oye Ekiti State Nigeria; ^7^ Biochemistry Unit, Department of Natural Sciences and Mathematics Williams V.S Tubman University Monrovia Republic of Liberia; ^8^ Institute of Drug Research and Development, SE Bogoro Center Afe Babalola University Ado‐Ekiti Ekiti State Nigeria; ^9^ Phytomedicine, Biochemical Toxicology and Biotechnology Research Laboratories, Department of Biochemistry, College of Sciences Afe Babalola University Ado‐Ekiti Ekiti State Nigeria; ^10^ Biotechnology and Structural Biology (BSB) Group, Department of Biochemistry and Microbiology University of Zululand KwaDlangezwa KwaZulu‐Natal South Africa; ^11^ Departmentof Physiology, Biochemistry and Pharmacology, College of Veterinary Medicine University of Kirkuk Kirkuk Iraq

**Keywords:** antioxidative, dietary, hepatoprotective, *Parkia biglobosa*, supplementation, *Zingiber officinale*

## Abstract

This study evaluated the effects of ginger (*Zingiber officinale*) and locust bean (*Parkia biglobosa*) dietary supplementation on liver function indices and the expression of apoptosis and inflammation. Sixty‐four female Wistar rats were randomly divided into eight groups and fed diets supplemented with graded amounts of ginger and locust beans for 8 weeks. Hepatic function parameters and oxidative stress biomarkers (GGT, AST, ALT, ALP, albumin, bilirubin, SOD, CAT, GPx, GSH, MDA, IL‐10, and TNF‐α) were comprehensively evaluated. Furthermore, hepatic gene expression analysis was performed to assess caspase‐3 and Bcl‐2. The results showed that diets supplemented with graded levels of ginger and locust beans significantly improved liver enzyme activities and modulated albumin and bilirubin levels. In addition, the supplemented diets decreased lipid peroxidation, reduced caspase‐3 expression, and increased Bcl‐2 expression. These findings suggest that ginger and locust beans possess significant hepatoprotective and anti‐inflammatory properties.

## Graphical abstract

Dietary supplementation with ginger (*Zingiber officinale*) and locust bean (*Parkia biglobosa*) for 55 days modulated hepatic function, oxidative stress, inflammatory markers, and apoptosis‐related gene expression in Wistar rats. Treatment increased liver function enzymes (GGT, AST, ALT, ALP), albumin, and antioxidant defenses (SOD, CAT, GPx, GSH), while reducing bilirubin, MDA, IL‐10, and TNF‐α levels. Furthermore, supplementation downregulated hepatic caspase‐3 expression and upregulated Bcl‐2 expression, indicating attenuation of apoptosis and enhanced hepatocellular protection.

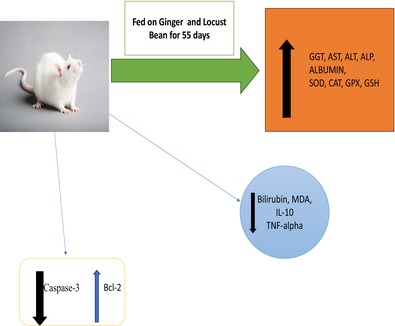

## 1 Introduction

Liver diseases constitute a major global health burden, arising from diverse etiologies such as viral infections, excessive alcohol consumption, and metabolic disorders, all of which can culminate in liver injury and failure (Gan et al. 2025; World Health Organization 2019). Given the liver's central role in metabolism, detoxification, and the synthesis of essential proteins, there is a pressing need to identify effective therapeutic and preventive strategies capable of mitigating hepatic damage and promoting liver health. Among the various interventions explored, dietary supplementation with natural bioactive compounds has attracted considerable attention due to their therapeutic potential and relatively low risk of adverse effects (Hernández‐Aquino and Muriel 2018; Narayanankutty et al. 2024).

In Nigeria, commercially available seasoning cubes are widely used as culinary flavor enhancers and have become indispensable in many households. These products typically contain high levels of sodium, starch, vegetable oils, and flavor enhancers such as monosodium glutamate (MSG). Excessive consumption of such ingredients has been associated with potential health risks, particularly hypertension and related metabolic complications. Consequently, there is growing interest in healthier, locally sourced alternatives prepared from natural spices, such as ginger and locust bean, especially in western regions of Nigeria where their traditional use is well established.

Ginger ( *Zingiber officinale* ) is a well‐known medicinal plant extensively studied for its antioxidant, anti‐inflammatory, and hepatoprotective properties (Ayustaningwarno et al. 2024; Mao et al. 2019). Its bioactive constituents, including gingerols, shogaols, and paradols, have been shown to exert protective effects by attenuating oxidative stress and inflammatory pathways—key contributors to liver injury (Habib et al. 2015). Notably, Mao et al. (2019) demonstrated that ginger supplementation significantly reduced hepatic oxidative stress markers and improved liver function indices in experimental animal models, highlighting its potential utility in liver disease management.

Similarly, locust bean ( *Parkia biglobosa* ) is a traditional African food condiment and medicinal resource with considerable nutritional and therapeutic value. It is rich in proteins, carbohydrates, essential vitamins, and bioactive compounds such as flavonoids, tannins, and polyphenols (Alawode et al. 2017; Komolafe et al. 2024). Previous studies have reported that locust bean exhibits antioxidant and anti‐inflammatory activities that may confer protection against hepatic injury. For example, Alawode et al. (2017) reported that locust bean extract ameliorated chemically induced liver toxicity, underscoring its potential role as a functional dietary supplement for liver health.

Apoptosis, or programmed cell death, is a fundamental biological process involved in maintaining hepatic cellular homeostasis and eliminating damaged hepatocytes. Caspase‐3 and B‐cell lymphoma‐2 (Bcl‐2) are key regulators of apoptotic signaling pathways in the liver. Caspase‐3 serves as a critical executioner enzyme in apoptosis, facilitating the removal of injured or dysfunctional hepatocytes (Hussar 2022; Mohamed and Magdy 2017). In contrast, Bcl‐2 is an anti‐apoptotic protein that promotes cell survival by inhibiting the mitochondrial apoptotic pathway (Kale et al. 2018). Dysregulation of these apoptotic mediators has been implicated in the pathogenesis of chronic liver diseases and hepatocellular carcinoma (Zhang et al. 2016).

In parts of western Nigeria, there is a prevailing belief that diets supplemented with ginger and locust bean offer greater health benefits than commercially available seasoning cubes; however, scientific evidence supporting this claim remains limited. Given the potential long‐term health implications associated with frequent consumption of conventional seasoning cubes, this study was designed to evaluate the effects of ginger and locust bean supplementation on liver function, oxidative stress, inflammation, and apoptotic markers, with a view to assessing their suitability as healthier dietary alternatives in Nigerian households.

## 2 Materials and Methods

### 2.1 Materials

Ginger powder was obtained from Nao Supermarket, Ado‐Ekiti, Ekiti State, Nigeria. A notable Nigerian seasoning cube was obtained from Nao Supermarket, Ado‐Ekiti, Ekiti State, Nigeria. Locust beans were purchased from Oja Oba Market, Oye‐Ekiti, Ekiti, Nigeria. Basal diet was purchased from the poultry food store opposite Saint Augustine, Oye‐Ekiti, Ekiti, Nigeria.

### 2.2 Chemical Reagents and Enzyme Kits

Ethanol and phosphate buffer were obtained from Sigma, Aldrich, Germany, while all the reagents used were of analytical grade. Also, all the enzyme kits used were products of Randox Laboratory (Crumlin, UK).

### 2.3 Preparation of Diet Supplement

Locust beans were dried for 2 weeks to ensure even dryness. The dried locust beans were ground into a fine powder. The powdered locust beans were mixed with powdered ginger and basal diet in specific proportions, as specified in Table 1. An adequate volume of water was added to form a batter, which was subsequently processed into rat feed pellets. The pellets were then oven‐dried at 50°C for 3 days to ensure proper hardening and moisture removal. This was packed inside an airtight polytene bag and kept in a freezer.

**TABLE 1:** Diet formulation ratio (g).

Ingredient	Group 1	Group 2	Group 3	Group 4	Group 5	Group 6	Group 7	Group 8
Basal diet (BD)	+	+	+	+	+	+	+	+
Locust beans (LB)	−	5	−	−	2.5	5	10	20
Ginger (G)	−	−	5	−	2.5	2.5	5	10
Nigerian seasoning cube (NSC)	−	−	−	5	−	−	−	−

*Note:* +: Presence of the ingredient in the diet formulation. −: Absence of the ingredient in the diet formulation. 2.5, 5, 10, and 20 g indicated the locust beans and ginger proportions mixed with the basal diet (which contain carbohydrate, protein, fat, and oil, minerals) for the treatment groups.

### 2.4 Experimental Animals

Sixty‐four female Wistar rats (1 month old, weighing 65–100 g) were obtained from Ekiti State University Animal House, Ekiti State. The rats were divided into eight groups and housed in a standard laboratory setting with a room temperature of 22°C–20°C and a 12‐h light/dark cycle. The animals were acclimatized for 1 week before the experiment. This study was approved by the Ethical Committee of the Faculty of Science, FUOYE, with the ethics approval number FUOYEFSC 201122–REC2025/029.

### 2.5 Experimental Design

Rats were weighed accurately and divided into groups based on their body weight in line with Table 1.

- Group 1: Rats fed with basal diet (Normal Control) (BD).
- Group 2: Rats fed with basal diet + locust beans (BD + 5 g LB).
- Group 3: Rats fed with basal diet + ginger (BD + 5 g G).
- Group 4: Rats fed with Basal diet + Nigerian notable seasoning cube (BD + 5 g NSC).
- Group 5: Rats fed with Basal diet + locust beans + Ginger (BD + 2.5 g LB + 2.5 g G).
- Group 6: Rats fed with Basal diet + locust beans + Ginger (BD + 5 g LB + 2.5 g G).
- Group 7: Rats fed with Basal diet + locust beans + Ginger (BD + 10 g LB + 5 g G).
- Group 8: Rats fed with Basal diet + locust beans + Ginger (BD + 20 g LB + 10 g G).
- Rats were provided with specific diets for a duration of 8 weeks. Additionally, the doses used in this study were based on a preliminary study conducted by the authors.

### 2.6 Tissue Collection and Processing

On the 55th day of feeding, the animals were fasted for 12 h (by removing their feed) prior to sacrifice. Following this, the animals were sacrificed through cervical dislocation, and blood samples were obtained via cardiac puncture. The liver of each animal was carefully harvested and rinsed with phosphate buffer to remove residual blood. Excess fat attached to the organ was trimmed using tissue paper, and the liver was then dried with filter paper. Blood was collected into plain bottles, centrifuged at 1500 rpm for 10 min, and the decanted samples (serum) were kept in the refrigerator for different biochemical analyses. The liver was also homogenized and centrifuged at 3000 rpm for 20 min. The homogenate was used for analysis, too.

### 2.7 Biochemical Parameters Studied

#### 2.7.1 Liver Function Indices

The activities of gamma glutamate transferase (GGT), aspartate transaminase (AST), alanine transaminase (ALT), and the levels of albumin, direct and total bilirubin were determined by following the protocol outlined in the commercial kits used.

#### 2.7.2 Pro‐ and Anti‐Inflammatory Determination

The levels of tumor necrosis factor‐alpha and interleukin‐10 were determined using the methods described in the commercial ELISA kits.

#### 2.7.3 Evaluation of Redox Status Biomarkers

The method described by Misra and Fridovich (1972) was used to assay for the activity of superoxide dismutase (SOD). The method described by Beers and Sizer (1952) was used to assay for the activity of catalase. Glutathione peroxidase (GPx) was determined using the method of Pippenger et al. (1998). Tietze (1969) was used for the glutathione (GSH) assay. The malondialdehyde concentration was quantified using the method described by Nelson and Cox (2004).

### 2.8 Molecular Gene Expression Analysis of Caspase‐3 and βcl‐2

Total RNA was isolated from tissue samples using the Quick‐RNA MiniPrep Kit (Zymo Research) for both Caspase‐3 and βcl‐2gene expression studies. To remove any potential DNA contaminants, the samples were treated with DNase I (NEB, Cat: M0303S). Following this, RNA quantification was carried out at 260 nm, and the purity was assessed by measuring absorbance ratios at 260 and 280 nm using an A&E Spectrophotometer (A&E Lab, UK).

For the conversion of RNA to cDNA, 1 μg of DNA‐free RNA from each sample was used. The reverse transcription was performed using the ProtoScript II First‐Strand cDNA Synthesis Kit (New England BioLabs). The reaction conditions were as follows: 65°C for 5 min to denature secondary structures, 42°C for 1 h for cDNA synthesis, and 80°C for 5 min to terminate the reaction. This step generated the cDNA needed for the subsequent qRT‐PCR analysis.

Quantitative real‐time PCR (qRT‐PCR) was used to quantify the mRNA expression levels of Caspase‐3 and Bcl‐2. Specific primers were designed for each gene (Table 2), and the reactions were carried out using SYBR Green Master Mix (Applied Biosystems) on a StepOnePlus Real‐Time PCR System (Applied Biosystems). The PCR cycling conditions were set as follows: an initial denaturation at 95°C for 10 min, followed by 40 cycles of 95°C for 15 s for denaturation, and 60°C for 1 min for annealing and extension. Relative gene expression levels were determined using the 2^−ΔΔCt^ method, with GAPDH used as the internal control to normalize the data for both Caspase‐3 and Bcl‐2.

**TABLE 2:** Reverse and forward primers used.

Parameters	Forward primer	Revere primer
CASPASE 3	5′‐GAGCTTGGAACGCGAAGAAA‐3′	5′‐GAGTCCATCGACTTGCTTCCA‐3′
Bcl‐2	5′‐ACTCTGTGTGGATTGGTGGC‐3′	5′‐AGCTCAGTAACAGTCCGCCT‐3′
GAPDH	5′‐GCAAGGATACTGAGAGCAAGAG‐3′	5′‐CATCTCCCTCACAATTCCATCC‐3′

### 2.9 Statistical Analysis

Data were expressed as ± SEM (standard error of mean). At 95% confidence interval and considered statistically significant at *p* < 0.05 with the aid of ANOVA analysis and Tukey's multiple comparisons test. The statistical evaluation was carried out using GraphPad Prism version 8.

## 3 Results and Discussion

### 3.1 Effect of Graded Levels of Ginger and Locust Bean Diets on Liver Enzymes in Rats

**FIGURE 1:**
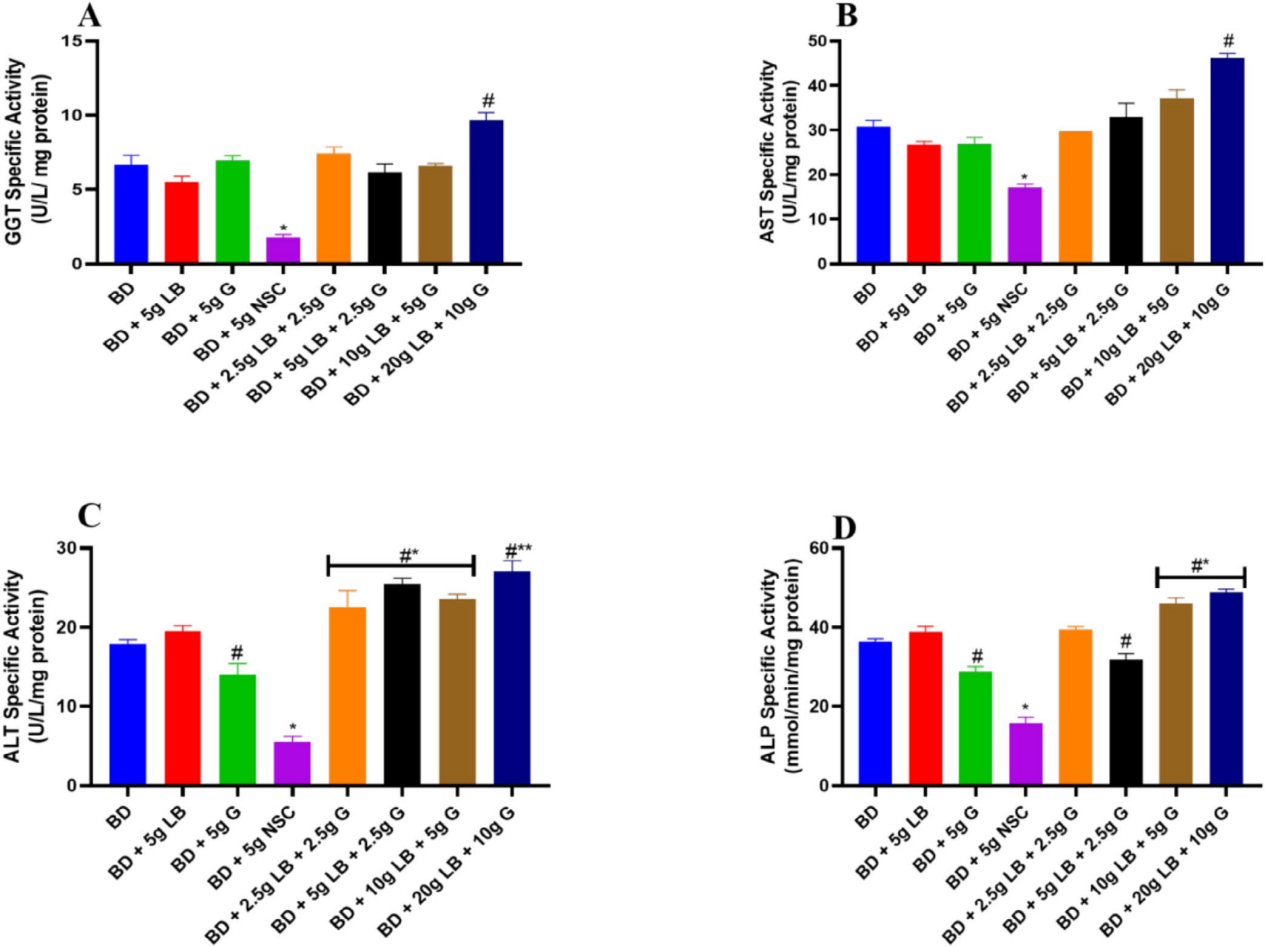
Liver enzyme activities of ginger and locust beans graded diets in rats. Each value is a mean of eight determinations ± SEM. # or * or #*p < 0.05 versus all the groups. (A) Gamma‐glutamyl transferase (GGT); (B) Aspartate aminotransferase (AST); (C) Alanine aminotransferase (ALT); and (D) Alkaline phosphatase (ALP).

The specific activity of gamma‐glutamyl transferase (GGT) was significantly (*p* < 0.05) decreased in the group that received a diet containing Nigerian seasoning cube compared to the BD group and the treated groups. However, the activity of this enzyme was significantly (*p* < 0.05) increased in all diets supplemented with ginger and locust beans compared to the Nigerian seasoning cube (Figure 1A).

Figure 1B, revealed that there was a significant (*p* < 0.05) decrease in the specific activity of aspartate aminotransferase in the group that was fed with a diet containing Nigerian seasoning cube when compared to the BD and other groups. It was also shown that the treated group (BD + 20 g LB + 10 g G) showed a significant (*p* < 0.05) increase in the specific activity of aspartate aminotransferase when compared to the BD group while the other treated groups have similar results to the BD group.

The specific activity of alanine aminotransferase (ALT) was significantly (*p* < 0.05) decreased in the group fed a diet containing Nigerian seasoning cube when compared to the BD and other groups (Figure 1C). The experimental rats placed on ginger and locust beans showed a significant (*p* < 0.05) increase in the specific activity of ALT when compared to the BD and Nigerian seasoning cube groups.

The activity of alkaline phosphatase (ALP) was significantly (*p* < 0.05) decreased in the group that received a diet containing the Nigerian seasoning cube compared to the BD and other groups (Figure 1D). In contrast, ALP activity was significantly (*p* < 0.05) elevated in all rats fed with graded diets of ginger and locust beans relative to the BD group.

The liver is a vital organ that performs essential functions, including nutrient metabolism, detoxification of harmful substances, bile production, synthesis of key proteins, nutrient storage, regulation of hormone levels, and maintenance of blood clotting mechanisms. Diet plays an important role in maintaining a healthy liver and in the progression of liver diseases (Jamioł‐Milc et al. 2023). Several natural products have shown protective effects on the liver due to their antioxidant and anti‐inflammatory properties (Li et al. 2024). This research investigated the effects of diets supplemented with graded levels of ginger and locust beans on liver indices such as GGT, aspartate aminotransferase (AST), ALT, ALP, albumin, and bilirubin (Figure 1).

In the current study, diets supplemented with ginger and locust beans improved the activity of GGT indicating their protective effect. GGT is an enzyme that facilitates the glutamyl cycle by catalyzing the transfer of gamma‐glutamyl groups from gamma‐glutamyl peptides, including GSH, to other peptides, amino acids, and water (Hoffman and Solter 2008). Serum GGT is expected to decrease the risk of clinical outcomes because the increase in GGT would lead to the increase of GSH (Lee et al. 2008). GSH is a major antioxidant that protects cells against oxidative stress by reducing H_2_O_2_ and scavenging reactive oxygen species and nitrogen radicals (Yuan and Kaplowitz 2009).

In this study, diets containing ginger and locust beans improved the activity of AST, supporting their protective effect. This aligns with a previous study that diet containing locust beans elevated AST level in rats (Iwuanyanwu et al. 2010). The reduction in AST activity by NSC indicates that there has been potential liver damage. AST plays a crucial role in amino acid metabolism as it facilitates the conversion of aspartate to α‐ketoglutarate, maintaining the balance of amino acids (Otto‐Ślusarczyk et al. 2016). It was also demonstrated that ginger and locust beans improved the activity of ALT in rats. ALT is an enzyme that catalyzes the conversion of alanine to pyruvate for cellular energy production (Gwaltney‐Brant 2016). NSC reduced the activity of ALT, thereby impairing the balance of amino acids and disrupting the production of energy.

This study also indicates that diets containing locust beans and ginger improved the activity of ALP. ALP is an enzyme that is important for various metabolic processes in the body, so a decrease in its activity by NSC could indicate potential metabolic disruption (Smith et al. 2020). This finding aligns with a previous study that locust beans and ginger might affect improving ALP activity (Doe and Martin 2022).

### 3.2 Effect of Ginger and Locust Beans Graded Diets on Albumin and Bilirubin Levels in Rats

**FIGURE 2:**
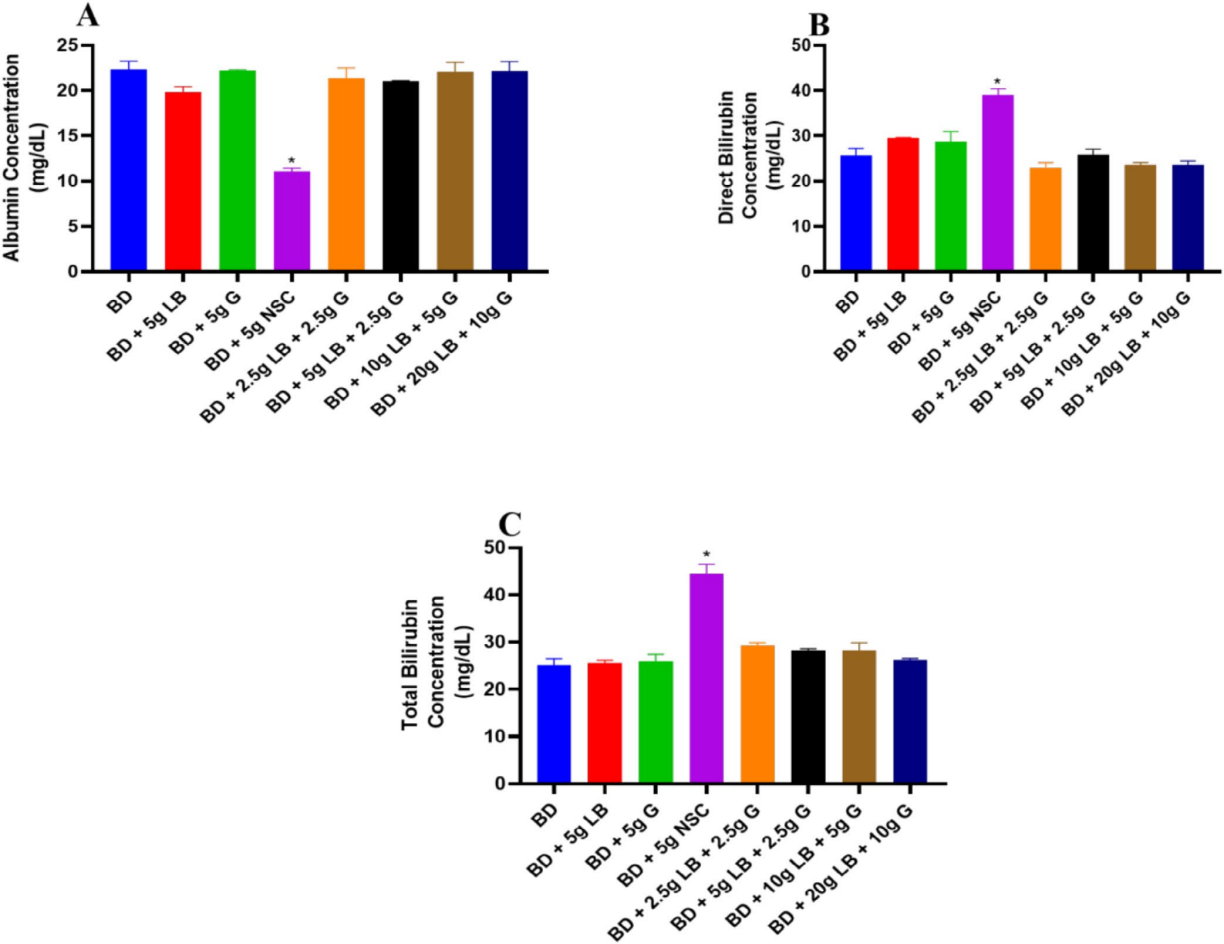
Bilirubin and albumin levels of ginger and locust beans graded diets in rats. Each value is a mean of eight determinations ± SEM. *p < 0.05 versus all the groups. (A) Albumin concentration; (B) Direct bilirubin concentration; and (C) Total bilirubin concentration.

The concentration of albumin was significantly (*p* < 0.05) reduced in the group fed with a diet containing Nigerian seasoning cube when compared to the BD and other experimental groups. All the animals placed on ginger and locust beans graded diets demonstrated a significant (*p* < 0.05) increase in the level of albumin compared with other groups (Figure 2A).

The result of the direct bilirubin levels in rats fed with graded diets of ginger and locust beans is shown in Figure 2B. The concentration of direct bilirubin was significantly (*p* < 0.05) elevated in the group fed with a diet containing Nigerian seasoning cube compared to the other groups. However, rats fed with graded levels of ginger and locust beans showed a significant (*p* < 0.05) decrease in direct bilirubin concentration compared to the Nigerian seasoning cube group.

The concentration of total bilirubin was significantly (*p* < 0.05) increased in the group fed with a diet containing Nigerian seasoning cube compared to other groups (Figure 2C). The animals fed on graded diets of ginger and locust beans showed similar results to the BD group.

This study also revealed that ginger and locust beans helped maintain normal albumin concentrations. In contrast, the significant (*p* < 0.05) decrease in albumin observed in the NSC group suggests potential liver damage. Similarly, ginger and locust beans preserved the levels of direct and total bilirubin, whereas NSC significantly (*p* < 0.05) increased them, indicating impaired bilirubin metabolism and possible liver injury. These findings are consistent with a previous report showing that ginger extract restored albumin and bilirubin levels in rats (Gholampour et al. 2017).

The outcomes of this study highlight the potential of ginger and locust beans in the improvement of liver function. Ginger and locust beans possess antioxidant, anti‐inflammatory, and hepatoprotective effects due to the presence of various bioactive components (Eboma et al. 2020; Mao et al. 2019). This study supports the growing evidence that bioactive compounds in ginger and locust beans can be utilized for improving liver health, as demonstrated in their ability to improve aspartate aminotransferase, GGT, ALT, ALP, albumin, and bilirubin (Figure 2).

### 3.3 Effect of Ginger and Locust Beans Graded Diets on Liver Redox Biomarkers in Rats

**FIGURE 3:**
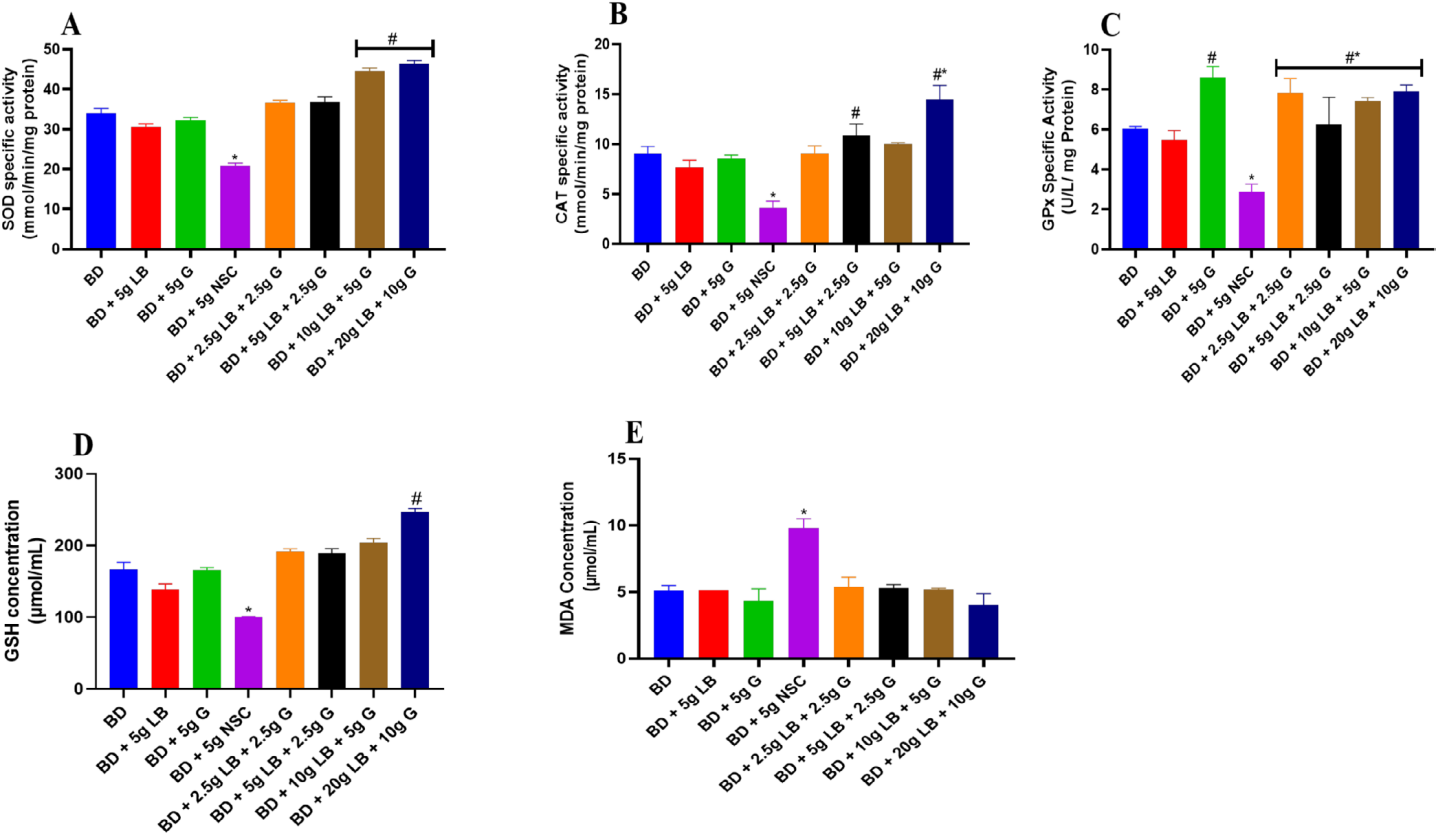
Effect of ginger and locust beans graded diets on liver redox biomarkers in rats. Each value is a mean of eight determinations ± SEM. * or #*p <* 0.05 versus all the groups. CAT, catalase; GPx, glutathione peroxidase; GSH, glutathione; MDA, malondialdehyde; SOD, super oxide dismutase. (A) Superoxide dismutase (SOD); (B) Catalase (CAT); (C) Glutathione peroxidase (GPx); (D) Reduced glutathione (GSH); and (E) Malondialdehyde (MDA).

The specific activity of SOD is shown in Figure 3A. SOD activity was significantly (*p* < 0.05) decreased in the group that received the diet containing Nigerian seasoning cube, compared with graded locust beans and ginger supplemented diets, as well as the basal diet. Rats receiving a diet supplemented with locust beans and ginger showed a significant (*p* < 0.05) increase in SOD activity compared with the Nigerian seasoning cube group.

The specific activity of catalase (CAT) was significantly (*p* < 0.05) decreased in the group that received the diet containing Nigerian seasoning cube when compared with locust beans and ginger supplemented diets, as well as the basal diet. However, rats receiving a diet supplemented with locust beans and ginger showed a significant (*p* < 0.05) increase in CAT specific activity compared to the Nigerian seasoning cube group (Figure 3D).

Gluthatione peroxidase (GPx) activity was significantly (*p* < 0.05) decreased in the group that received the diet containing Nigerian seasoning cube compared with locust beans, and ginger, supplemented diets and basal diet. Moreover, rats fed with diets supplemented with locust beans and ginger showed a significant (*p* < 0.05) increase in GPx activity (*p* < 0.05) compared to the Nigerian seasoning cube group (Figure 3C).

The GSH level was significantly (*p* < 0.05) decreased in the group that received the diet containing Nigerian seasoning cube compared to locust beans and ginger supplemented diets and basal diet. Hence, GSH level was significantly (*p* < 0.05) increased in groups placed on locust beans and ginger supplemented diets compared to the Nigerian seasoning cube group (Figure 3D).

The malondialdehyde (MDA) level was significantly (*p* < 0.05) increased in the group that received the diet containing Nigerian seasoning cube, compared with locust beans and ginger, supplemented diets and basal diet (Figure 3E). However, rats receiving a diet supplemented with locust beans and ginger showed a significant (*p* < 0.05) decrease in malondialdehyde (MDA) level compared to the Nigerian seasoning cube group.

The results of this study highlight the significant antioxidative and hepatoprotective effects of ginger (G) and locust beans (LB) in restoring and enhancing liver redox biomarkers in rats. Specifically, the improvements in SOD, catalase (CAT), GPx activities, as well as GSH level, coupled with the reduction in malondialdehyde (MDA) levels, demonstrate that these natural compounds effectively counteract oxidative stress induced by the Nigerian seasoning cube (NSC) diet. When compared to previous studies, these findings reinforce the therapeutic potential of dietary supplementation with natural antioxidants in mitigating liver damage (Figure 3).

As observed in the current study, reports significant increases in the activities of key antioxidant enzymes such as SOD, CAT, and GPx, as well as non‐enzymatic antioxidants, that is, GSH—upon supplementation with LB and G, reversing the deficits caused by the NSC diet. This is consistent with findings from a similar study by Mao et al. (2019), which demonstrated that ginger supplementation improved antioxidant enzyme activities in animal models subjected to oxidative stress. Mao et al. (2019) observed that ginger's bioactive components, particularly gingerols and shogaols, played a pivotal role in neutralizing ROS and reducing oxidative damage in liver tissues. Similarly, the polyphenolic content of locust beans, rich in flavonoids and tannins, has been shown to exert comparable antioxidant effects, as highlighted in a study by Alawode et al. (2017), where locust beans ameliorated chemically induced liver toxicity.

The enhancement of SOD activity observed in this study aligns with earlier research showing that ginger and locust beans effectively upregulate SOD, thus increasing the liver's capacity to dismutate superoxide radicals into less harmful molecules such as hydrogen peroxide. This is crucial, as the accumulation of superoxide anions can exacerbate oxidative stress, contributing to cellular damage. The synergistic effect of LB and G in boosting SOD levels beyond those seen in the basal diet group emphasizes the complementary nature of these supplements in oxidative stress management.

The significant restoration of CAT activity in the LB + G group corroborates with findings from Dongiovanni et al. (2015), who observed that diets rich in polyphenolic compounds could upregulate CAT activity, thereby reducing the harmful buildup of hydrogen peroxide. This mechanism is vital in preventing the formation of hydroxyl radicals, which are among the most reactive and damaging ROS. The enhanced CAT activity in the supplemented groups suggests that LB and G can provide a more comprehensive antioxidative defense compared to NSC alone.

The restoration of GPx activity in this study mirrors previous work by Ahmed et al. (2016), which highlighted the role of ginger in boosting GPx activity in oxidative stress‐induced liver injury. The ability of Locust beans and ginger to significantly elevate GPx levels suggests that these supplements improve the liver's ability to detoxify peroxides, preventing lipid peroxidation and subsequent cell membrane damage. This finding is particularly relevant, as impaired GPx activity is often associated with various liver diseases, including non‐alcoholic fatty liver disease (NAFLD) and alcohol‐induced liver injury. Additionally, the marked increase in reduced GSH levels following supplementation with Locust beans and ginger is consistent with the role of GSH as a crucial intracellular antioxidant that maintains redox balance and detoxifies harmful substances. A study by Brigelius‐Flohé and Maiorino (2013) emphasized the importance of maintaining adequate GSH levels to prevent oxidative damage and support liver function. The ability of LB and G to restore GSH levels beyond those in the NSC group highlights their potential to reverse oxidative stress‐related depletion of this key antioxidant. The synergy between LB's rich polyphenolic content and ginger's bioactive compounds likely plays a pivotal role in enhancing GSH synthesis and regeneration.

Malondialdehyde (MDA) is a byproduct of lipid peroxidation and serves as a marker of oxidative stress and cellular damage. The elevated levels of MDA in the NSC group suggest enhanced lipid peroxidation, consistent with studies that link high oxidative stress diets to increased MDA levels. However, the significant reduction in MDA levels in the Locust beans and ginger group indicates that these supplements effectively prevent lipid peroxidation, preserving membrane integrity. This finding is in agreement with earlier studies by Ayala et al. (2014), who demonstrated that the combination of natural antioxidants could reduce MDA levels and mitigate oxidative damage in liver tissues.

### 3.4 Effect of Ginger and Locust Beans Graded Diets on Liver Interleukin‐10 (IL‐10) and Tumor Necrosis Factor (TNF‐α) Levels in Rats

**FIGURE 4:**
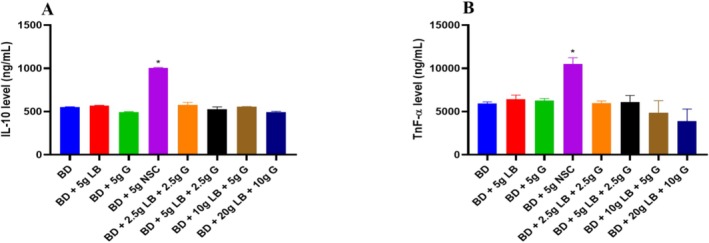
Effect of ginger and locust beans graded diets on liver interleukin‐10 and tumor necrosis factor in rats. Each value is a mean of eight determinations ± SEM. *p < 0.05 versus all the groups. (A) Interleukin‐10 (IL‐10); and (B) Tumour necrosis factor‐α (TNF‐α).

The interleukin‐10 (IL‐10) level was significantly (*p* < 0.05) increased in the group that received the diet containing Nigerian seasoning cube compared to other groups (Figure 4A). However, rats receiving a diet supplemented with locust beans and ginger showed a significant (*p* < 0.05) decrease in interleukin‐10 (IL‐10) levels (*p* < 0.05) compared to the Nigerian seasoning cube group.

The tumor necrosis factor (TNF‐α) levels were significantly (*p* < 0.05) increased in the group that was placed on a diet containing Nigerian seasoning cube, compared to other groups. However, rats receiving a diet supplemented with locust beans and ginger showed a significant (*p* < 0.05) decrease in tumor necrosis factor (TNF‐α) levels when compared to the Nigerian seasoning cube group (Figure 4B).

In comparing the findings of this study with previous research, it is evident that the combination of locust beans and ginger offers a broad spectrum of antioxidative and hepatoprotective benefits. Studies by Habib et al. (2015) and Adedayo et al. (2014) also reported that the anti‐inflammatory and antioxidative properties of ginger and locust beans extend beyond their ability to enhance enzyme activities. These studies demonstrated that both LB and G could modulate inflammatory pathways, reducing the expression of pro‐inflammatory cytokines such as TNF‐α and increasing anti‐inflammatory markers like IL‐10, thereby preventing liver damage induced by oxidative stress.

In addition to their antioxidant properties, ginger (G) and locust beans (LB) also exert significant effects on inflammatory markers, particularly interleukin‐10 (IL‐10) and tumor necrosis factor‐alpha (TNF‐α). These cytokines play crucial roles in regulating inflammation, which is a key driver in the pathogenesis of many liver diseases, including non‐alcoholic fatty liver disease (NAFLD), alcoholic liver disease (ALD), and other chronic liver conditions.

IL‐10 is an anti‐inflammatory cytokine that plays a vital role in regulating immune responses and mitigating excessive inflammation. It acts by inhibiting the synthesis of pro‐inflammatory cytokines, such as TNF‐α, IL‐1, and IL‐6, thus limiting tissue damage caused by chronic inflammation. In this study, the levels of IL‐10 were significantly elevated in the group fed the Nigerian seasoning cube (NSC) diet, likely as a compensatory response to increased inflammation induced by the high‐oxidative stress diet. This finding is consistent with previous studies showing that heightened inflammatory conditions can trigger an upregulation of IL‐10 as the body attempts to counteract inflammation and limit tissue damage (Habib et al. 2015).

TNF‐α is a major pro‐inflammatory cytokine that is involved in systemic inflammation and plays a critical role in the pathogenesis of liver injury. It is produced primarily by macrophages and hepatocytes in response to stress, infection, or injury and is known to exacerbate liver damage by promoting inflammation, hepatocyte apoptosis, and fibrosis. Elevated levels of TNF‐α are often associated with the progression of chronic liver diseases, including hepatitis, cirrhosis, and hepatocellular carcinoma (HCC). In this study, TNF‐α levels were significantly increased in rats fed the NSC diet, which is consistent with the pro‐inflammatory nature of such diets. This elevation in TNF‐α likely reflects the liver's response to increased oxidative stress and damage induced by the high‐oxidative stress ingredients in the NSC diet. The overproduction of TNF‐α can lead to the activation of inflammatory pathways, contributing to the recruitment of immune cells and further exacerbating liver damage. Remarkably, supplementation with LB and G significantly reduced TNF‐α levels, restoring them to levels comparable to the BD group. This finding suggests that ginger and locust beans possess strong anti‐inflammatory properties, capable of downregulating the expression of pro‐inflammatory cytokines such as TNF‐α. Previous studies by Oboh et al. (2018) and Alawode et al. (2017) have shown that the polyphenols in locust beans and the bioactive compounds in ginger can inhibit the activation of nuclear factor‐kappa B (NF‐κB), a transcription factor that regulates TNF‐α expression. By inhibiting NF‐κB activation, LB and G reduce the production of TNF‐α, thus alleviating inflammation and protecting against liver injury.

When compared to previous studies, the effects of LB and G on IL‐10 and TNF‐α align with the well‐documented anti‐inflammatory properties of these supplements. For instance, the study by Adedayo et al. (2014) highlighted the role of polyphenols in locust beans in modulating immune responses and reducing pro‐inflammatory cytokine production. Similarly, Habib et al. (2015) reported that ginger supplementation could downregulate TNF‐α levels in animal models of liver injury, providing further evidence of the anti‐inflammatory effects observed in this study.

### 3.5 Effect of Ginger and Locust Beans Graded Diets on Caspase‐3 and Beta‐Cell Lymphoma‐2 (βcl‐2) in Rats

**FIGURE 5:**
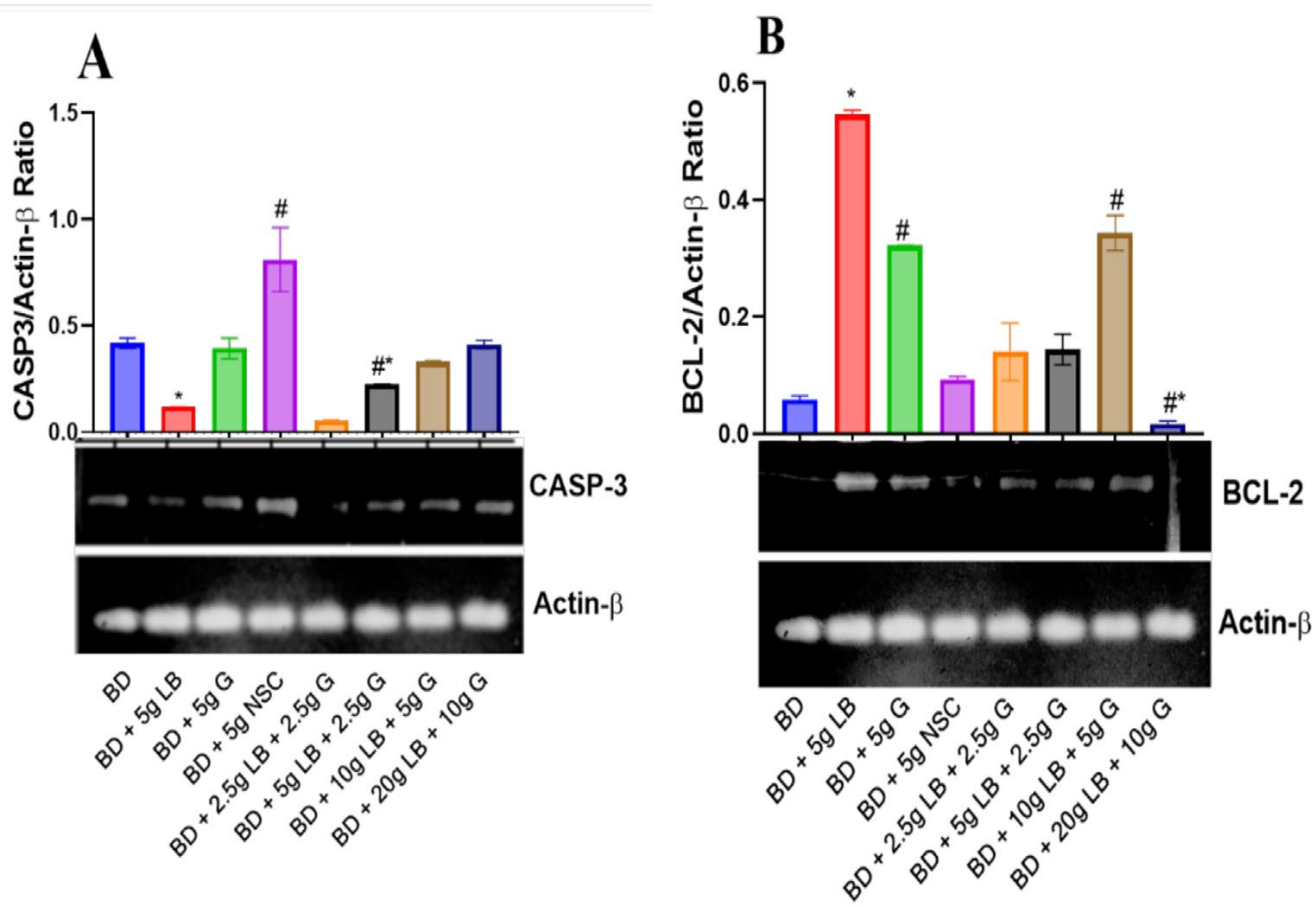
Effect of ginger and locust beans graded diets on relative gene expression of caspase‐3 and beta‐cell lymphoma‐2 (βcl‐2) in rats. Each value is a mean of eight determinations ± SEM. # or * or #*p < 0.05 versus all the groups. (A) Caspase‐3; and (B) β‐cell lymphoma‐2 (Bcl‐2).

The caspase‐3 expression was significantly (*p* < 0.05) increased in the group that received the diet containing Nigerian seasoning cube, compared to the locust beans and ginger supplemented groups (Figure 5A). But the animals placed on a locust beans and ginger supplemented diet showed a significant (*p* < 0.05) decrease in caspase‐3 expression compared to the Nigerian seasoning cube group.

The beta‐cell lymphoma‐2 (βcl‐2) expression was significantly decreased (*p* < 0.05) in the group that received the diet containing Nigerian seasoning cube, compared to other groups (Figure 5B). However, rats receiving a diet supplemented with locust beans and ginger showed a significant increase (*p* < 0.05) in beta‐cell lymphoma‐2 (βcl‐2) expression compared to the Nigerian seasoning cube group.

An increased expression of caspase‐3 in the liver suggests that hepatocytes (liver cells) are undergoing increased apoptosis, or programmed cell death, as observed in the group that was supplemented with NSC. Caspase‐3 is an executioner caspase, meaning its activation is a crucial step in the cell's self‐destruction process (Shi et al. 2024). Hence, the significantly elevated expression of caspase‐3 is an important indicator of cellular injury and points toward several potential underlying conditions. However, the expression of caspase‐3 was reduced in diets supplemented with LB and G, which indicates their potential protective effects against liver damage. This effect may be attributed to the presence of bioactive compounds such as flavonoids, tannins, and phenolic compounds, which are known to modulate apoptotic pathways, reduce oxidative stress, and promote hepatocellular protection.

The results presented in this study revealed significant expression of beta‐cell lymphoma‐2 (βcl‐2), a crucial anti‐apoptotic protein, in rats fed with diets supplemented with ginger (G) and locust beans (LB). βcl‐2 plays a central role in regulating apoptosis by inhibiting the mitochondrial pathway of cell death, promoting cell survival, and maintaining tissue homeostasis. The observed changes in βcl‐2 expression highlight the potential protective effects of LB and G against liver damage. In this study, the expression of Bcl‐2 was significantly reduced (*p* < 0.05) in the group receiving the Nigerian seasoning cube (NSC) diet supplemented with locust beans and ginger compared to the group receiving only the basal diet (BD). This reduction in βcl‐2 expression suggests that the NSC diet may contribute to increased susceptibility to apoptosis and liver cell damage, likely due to its oxidative stress‐inducing properties. As βcl‐2 inhibits apoptosis by binding to pro‐apoptotic proteins, its down regulation could indicate a weakened defense against cell death, further exacerbating liver injury.

Also, rats receiving LB and G supplementation showed a marked increase in βcl‐2 expression compared to the NSC group, reversing the decline observed. This increase in βcl‐2 expression suggests that LB and G supplementation promotes cell survival by preventing apoptosis and protecting liver cells from damage. These findings are consistent with previous studies showing that bioactive compounds with strong antioxidant and anti‐inflammatory properties, including gingerols from ginger and polyphenols from locust beans, can enhance Bcl‐2 expression and suppress apoptosis under oxidative stress. Such as Cui et al. (2018), which highlighted the protective effects of ginger and polyphenol‐rich diets on liver cells by up‐regulating βcl‐2 and inhibiting apoptosis. The current study further demonstrates that the combination of LB and G offers a synergistic effect, enhancing the liver's defense mechanisms by increasing βcl‐2 expression and promoting cellular survival.

The results also suggest that the LB and G combination can outperform the basal diet alone in maintaining Caspase‐3 and βcl‐2 expressions. This finding highlights the potential of LB and G as dietary supplements to prevent liver cell apoptosis, especially in the context of diets that induce oxidative stress and inflammation, such as those containing NSC.

## 4 Conclusion

The present study provides strong evidence that dietary supplementation with ginger and locust beans improves liver function indices; these dietary interventions modulated critical apoptotic pathways, as reflected by the suppression of Caspase‐3 expression and the enhancement of Bcl‐2 expression. Collectively, these findings suggest that ginger and *locust beans* help preserve hepatocyte integrity by preventing apoptosis, thereby contributing to overall liver protection. Further research is recommended to elucidate the underlying molecular mechanisms and to explore their potential applications in managing liver disorders.

## Author Contributions

All authors were involved in designing, conducting the experiment, analyzing the data, preparing this manuscript, and approving this manuscript for submission to this your reputable journal.

## Funding

This work was self‐funded.

## Conflicts of Interest

The authors declare no conflicts of interest.

## Data Availability

The data that support the findings of this study are available on request from the corresponding author. The data are not publicly available due to privacy or ethical restrictions.
